# Bioactive Peptides from Algae: Traditional and Novel Generation Strategies, Structure-Function Relationships, and Bioinformatics as Predictive Tools for Bioactivity

**DOI:** 10.3390/md20050317

**Published:** 2022-05-10

**Authors:** Jack O’Connor, Marco Garcia-Vaquero, Steve Meaney, Brijesh Kumar Tiwari

**Affiliations:** 1 School of Biological & Health Sciences, Technological University Dublin, Dublin 2, Ireland; jack.oconnor@teagasc.ie (J.O.); steve.meaney@tudublin.ie (S.M.); 2 Department of Food Chemistry and Technology, Teagasc Food Research Centre, Ashtown, Dublin 15, Ireland; brijesh.tiwari@teagasc.ie; 3 Section of Food and Nutrition, School Agriculture and Food Science, University College Dublin, Belfield, Dublin 4, Ireland

**Keywords:** in silico, biotechnology, cryptides, anti-hypertensive, antioxidant, anti-cancer

## Abstract

Over the last decade, algae have been explored as alternative and sustainable protein sources for a balanced diet and more recently, as a potential source of algal-derived bioactive peptides with potential health benefits. This review will focus on the emerging processes for the generation and isolation of bioactive peptides or cryptides from algae, including: (1) pre-treatments of algae for the extraction of protein by physical and biochemical methods; and (2) methods for the generation of bioactive including enzymatic hydrolysis and other emerging methods. To date, the main biological properties of the peptides identified from algae, including anti-hypertensive, antioxidant and anti-proliferative/cytotoxic effects (for this review, anti-proliferative/cytotoxic will be referred to by the term anti-cancer), assayed in vitro and/or in vivo, will also be summarized emphasizing the structure–function relationship and mechanism of action of these peptides. Moreover, the use of in silico methods, such as quantitative structural activity relationships (QSAR) and molecular docking for the identification of specific peptides of bioactive interest from hydrolysates will be described in detail together with the main challenges and opportunities to exploit algae as a source of bioactive peptides.

## 1. Introduction

The world’s population is estimated to reach 9 billion by 2050 and there is continuing pressure on the current agricultural and food production systems to meet the increased food demands without damaging the environment [1,2]. Algae (macro- and microalgae) have become an attractive source of raw protein for the food industry [3] due to a potential protein content which often exceeds that of other common protein sources such as milk and soy (which contain approximately 40% protein on a dry weight basis) [4]. For example, the dry weight yield of protein in dried algal biomass can be up to 47% in the macroalgae *Pyropia* sp. [5] or 65% in the microalga *Chorella* sp. [6]. Thus, the production of algae offers excellent opportunities to increase food production without increasing deforestation or encroaching upon natural habitats while benefitting from an all-year harvest for algae [3]. In contrast to protein from milk (0.13 ton/acre/annum) and soybean (0.41–0.81 ton/acre/annum), macro and microalgae biomass can produce high protein yields ranging between 1.62 and 6.1, and 4.1 and 7.3 ton/acre/annum for macroalgae and microalgae, respectively [7]. 

Algal proteins are also a source of bioactive peptides, also named cryptides, as these compounds have the ability to exert direct physiological effects once they are released from their parent proteins where they remain inactive [8]. Similar to endogenous peptide hormones (e.g., those derived from proopiomelanocortin, insulin and angiotensin), bioactive peptides derive from parent polypeptide sequences through a series of controlled and specific proteolytic cleavages [9]. In some cases, several different sequences with a hormone-like action can be derived from the same parent polypeptide through differential cleavage [9]. Although numerous bioactive peptides with potential health benefits, e.g., anti-hypertensive properties, have been isolated from macro- and microalgae, there are technical challenges associated with the production and commercialization of bioactive peptides that still need to be addressed. 

This review aims to provide a comprehensive summary of the main approaches for the generation and isolation of bioactive peptides from algae. The review will focus mainly on novel uses of pre-treatment methods for the extraction of protein from algal biomass by physical and biochemical methods, as well as the enzymatic hydrolysis and other emerging methods for the release of bioactive peptides from algae. The main biological properties of these peptides (anti-hypertensive, antioxidant, and anti-cancer) and the structure–function relationships of known peptide sequences from algae will also be discussed in relation to their hypothesized mechanisms of action. Moreover, the recent developments in bioinformatics or in silico tools helping in the identification of these structures and their health benefits will also be discussed together with the main challenges and opportunities of bioactive peptides from algae.

## 2. Process of Generation and Isolation of Bioactive Peptides

The initial extraction of protein from raw biomass by the use of pre-treatments is needed as the first step for further protein processing for the generation and subsequent isolation of bioactive peptides.

### 2.1. Pre-Treatments of the Algal Biomass

Although algae are described in the literature as biomass rich in proteins, the complex structures of the carbohydrate-rich algal cell walls prevent immediate access to these compounds, and thus, algae must be pre-treated by either physical or biochemical methods to allow the release of proteins from the biomass [10]. Amongst all the physical methods available, the application of physical methods, such as pulsed electric fields (PEF), ultrasound-assisted extraction (UAE) and microwave-assisted extraction (MAE), has shown promising results in algae. Other enzyme-based methods use the application of high specific-activity enzyme preparations to degrade the cell walls [11]. The specific pre-treatments must be tailored and optimized on the basis of the algal species studied in order to achieve high yields of proteins. For example, the macroalgae *Chondrus crispus* has a cellulose microfibril base cell wall and a carrageenan matrix and thus, the extraction of protein from this macroalgae requires an enzymatic mixture with high carrageenase and cellulase activities [12,13].

#### 2.1.1. Physical Pre-Treatments

PEF is based on exposing cells to a high-strength electric field, inducing the formation of transient pores in the cell walls in a process known as electroporation [14]. The disruption of the cell walls by PEF can also lead to the formation of permanent or transient pores, allowing the mass transfer of small molecular weight compounds including protein extraction [15] to the outside of the cells and into the solution [16]. Pore sizes may also be further influenced by PEF duration, for instance, the pore radius for short pulses (5 μs) was approximately 0.8–1.6 nm, while when using longer pulses (10 μs) the pore radius increased up to at least approximately 5 nm, which was considered as irreversibly formed [17]. To date, PEF has been primarily used as a method to extract lipids from algae in connection with biofuel manufacture [17]. Vanthoor-Koopmans, Wijffels, Barbosa and Eppink [4] reported that small peptides and free amino acids present in the cell or created during the PEF process may be released from the biomass following PEF, while larger peptides and proteins were retained within the cell. Thus, according to the authors, adding a protein solubilization step will benefit these extraction processes. This solubilization can be achieved by adding surfactants and/or increasing the pH to untangle proteins, increasing their solubilization and extraction ratios [4,18]. The yield of the extracted protein from *Nannochloropsis* spp. doubled when using alkaline solvents (pH 8) compared to water; however, the recovery of proteins from the biomass was still low compared to other physical extraction procedures, such as UAE [19]. PEF has been described as a promising method for the extraction of proteins from the macroalga *Ulva ohnoi* [20]. The authors achieved a 3-fold increase in the yield of proteins from *U. ohnoi* of with the application of PEF. Similarly, in the case of *Arthrospira platensis*, a microalgae species with high protein content, PEF treatment followed by soaking the biomass in water increased the extraction of the intracellular proteins (c-phycocyanin) 12.7-fold compared to the control [21]. Parniakov, Barba, Grimi, Marchal, Jubeau, Lebovka and Vorobiev [19] emphasized the advantages of PEF in relation to power usage and cost-effectivity of the treatments, although the yields of protein extraction were lower compared to UAE pre-treatments. The power consumption of PEF was approximately 100 kJ/kg at 20 kV/cm for 6 ms, while in the case of UAE it was 250 kJ/kg at 200 W for 10 min [19].

UAE is based on the generation of high-frequency ultrasonic waves in a liquid, which generates a great number of bubbles that collapse (cavitation) and release a burst of energy that can disrupt the algal cell walls [22]. Advantages of this technology include the possibility to combine its application with various solvents, targeting the extraction of different compounds from the biomass, as well as an increased extraction efficiency in terms of reduced times and energy of extraction compared to conventional solvent extraction processes [23]. For example, the use of UAE with a subsequent alkaline treatment allowed the extraction of 57% of the total proteins from *Ascophyllum nodosum* [24]. Moreover, the use of ultrasounds enabled 2.29-fold higher yields of protein extraction compared to conventional methods from the microalga *A. platensis* [25]. 

MAE has also been used to extract protein from algae [26]. MAE uses oscillating electric fields which cause vibrational friction of polar molecules in the cells, which allows heating of the sample to occur. The main advantage of MAE is that a small amount of or even no solvents are required, as well as the fast extraction times of this technique compared to conventional methods [27,28]. Juin, Chérouvrier, Thiéry, Gagez, Bérard, Joguet, Kaas, Cadoret and Picot [26] used MAE to extract water-soluble proteins from *Porphyridium purpureum*, focusing mainly on the extraction of phycobilin proteins. The authors reported that MAE improves the extraction yields of compounds and significatively reduced the time of extraction to seconds compared to the hours needed to generate similar extraction yields when using traditional solving extraction methods. Moreover, MAE appeared to be more efficient in protecting thermolabile compounds during the process of extraction, as the application of high temperatures for prolonged periods of time needed during conventional solvent extraction may have a negative impact on these compounds once they are extracted [29].

#### 2.1.2. Enzymatic Pre-Treatments

One of the key advantages of enzymatic pre-treatments over other mechanical methods is the relatively low temperatures required, allowing the release of both peptides and proteins from algae with minimal or no damage to their structures [30]. However, the high variability of the composition of the algal cell walls between species requires will require the customization and optimization of these enzymatic treatments [11]. For example, a mixture of trypsin, collagenase, lysozyme, and autolysin was useful as a pre-treatment to disrupt the cell walls of *Chlamydomonas reinhardtii* [31]. Autolysin was the most efficient enzyme as an enzymatic treatment and showed to be preferable to other chemical and mechanical methods such as solvents and sonication [31]. Moreover, the authors also reported that longer pre-treatments resulted in the total lysis of the cells advantageously resulting in increased extraction yields of cellular compounds measured as proteins and lipids [31]. Other pre-treatments using cellulase followed by protein hydrolysis with bromelain were effective when extracting proteinaceous concentrates from *Fucus spiralis*, increasing its extraction yields by 1.5-fold compared to those achieved by using bromelain alone [32]. The protein content of *Macrocystis pyrifera* and *Chondracanthus chamissoi* was increased by disrupting the carbohydrate matrix of the algae by using cellulase, increasing the yields of protein extraction [33]. As expected, optimum enzymatic conditions also varied between both macroalgae, achieving protein extraction yields of approximately 75% from *M. pyrifera* by an optimized enzymatic treatment with cellulase (1:10, enzyme:seaweed ratio) for 18 h and yields of 36% from *C. chamissoi* using the same enzyme:seaweed ratio for 12 h [33]. Moreover, Fleurence, Massiani, Guyader and Mabeau [13] reported that a mix of carrageenase/cellulase has a 10-fold higher extraction efficiency over the use of just carrageenase alone in *C. crispus*. Similarly, mixtures of agarase/cellulose achieved a 3-fold increase in protein extraction from *G. verrucosa*, while in the case of *P. palmata,* the combined xylanase/cellulase had similar protein extraction yields to those of control [13]. 

### 2.2. Generation of Bioactive Peptides

Once the protein is extracted from the biomass, the classical method for the generation of bioactive peptides uses proteases to break peptide bonds and generate hydrolysates containing a complex mix of peptides [34]. Enzymes exert their action by cleaving sequence motifs within a protein. The preferred cleavage sites of each of these enzymes are summarized in Table 1.

Amongst all the proteases, trypsin, chymotrypsin and pepsin have been the most widely used enzymes for the generation of bioactive peptides. Trypsin has a specific binding affinity for positively charged side chains of the amino acids lysine and arginine. Trypsin’s cleavage site is on the C-terminal side of the amino acid residues. Hydrolysis is decreased with the presence of acidic amino acids on either side of the cleavage site. Cleavage will not occur if a proline residue is present on the carboxyl side of the cleavage site [35]. Chymotrypsin is another serine protease which is itself activated by trypsin cleaving the bond at residues 15 and 16 (arginine and isoleucine). The chymotrypsin enzyme catalyzes the hydrolysis of proteins by cleaving the molecules at hydrophobic amino acid residues, such as the L-isomers of tyrosine, phenylalanine, and tryptophan. It also has the capability of acting on amides and esters of susceptible amino acids [47]. The use of these enzymes can result in the one key advantages of this approach, which is the reproducibility of the process such that similar proteins and hydrolysates products are generated.

Emerging technologies have also been used to hydrolyze protein extracts and generate bioactive peptides from algae and other food products and by-products [48,49]. Amongst them, subcritical water (SCW) processing has been gaining attention as both a green extraction and protein hydrolysis method [50,51,52]. SCW does not require the use of expensive and lengthy reaction times and it can be a method of extracting compounds from highly insoluble media which are ecologically damaging by-products from industries, such as poultry waste [53], hog fur [54] and fisheries waste [55]. In fact, SCW production of bioactive peptides also significantly reduces the processing time of hydrolysis of collagen by a factor of almost 300 when using enzymatic methods such as collagenase [56] to up 5 min [57]. The use of SCW to produce amino acids and peptides from waste and under-utilized by-products could give these industries a new revenue stream while mitigating the ecological and economic issues currently associated with the disposal of these by-products [58]. 

The SCW process maintains water in subcritical conditions inside the reaction chamber by using oven temperatures ranging from >100 °C to <374 °C and an internal pressure of <22 MPa, stimulating the formation of hydronium (H_3_O^+^) and hydroxide ions (HO^−^) that allow water to interact as a basic or acidic catalyst [52]. The pressure applied during SCW will cause the unfolding and loss of secondary, tertiary, and quaternary structures of the protein, while the ions will interact with the amino acids [49]. The amino acids that are particularly vulnerable to hydrolysis by SCW are aspartic acid and glutamic acid, affected by the weak acidic conditions as their carboxyl group becomes a proton donor for the hydrolysis of the peptide bond next to it [52]. Ahmed, Mulla, Al-Ruwaih and Arfat [57] reported that using sequential pressure pretreatment of 300 MPa for 15 min increased the degree of hydrolysis for proteins when being hydrolyzed with alcalase [57], an enzyme that cleaves the carboxyl side of the amino acids E, L, Y, Q and E [58]. This indicates that the application of high pressure leads to a certain degree of protein unfolding, potentially increasing access of the enzyme to substrate cleavage sites [57]. A similar study using soy protein performed by Meinlschmidt et al. [59] showed similar enhanced digestibility following exposure to a pressure of 100 MPa in the presence of the enzyme flavourzyme for 15 min. Under these conditions, when the pressure exceeds 100 MPa the enzyme itself starts to become denatured by the pressure, and its activity is lost [59].

SCW appears to have some cleavage specificity for bonds adjacent to aspartyl residues, with some 44% of the peptides produced from subcritical water-mediated hydrolysis of BSA containing an N-terminal aspartic [52]. Moreover, peptide production from the microalgae *A. platensis* was optimal at 160 °C, while temperatures of over 220 °C produced an intense degradation of these proteins and the release of free amino acids rather than peptides, with no distinguishable bands when analyzing the hydrolysates by denaturing protein electrophoresis [49,55]. SCW has also been explored for the production of amino acids at temperatures of 240 °C [60,61], while temperatures reaching 260 °C will result in the degradation of amino acids to organic acids and ammonia [61]. These data illustrate the need for careful control of temperature during SCW processing to ensure the appropriate release of protein and peptides rather than terminal degradation.

After the proteins have been processed and hydrolyzed to generate bioactive peptides, one or several purification processes are frequently applied to isolate these molecules further. Overall, most authors used one or several steps of molecular weight cut-off filtration (MWCO) to fractionate the compounds of the hydrolysates based on their molecular weight [62]. Thereby, Megías et al. [63] used 5 kDa membranes to remove, concentrate and purify peptides in the hydrolysate by removing larger unhydrolyzed protein fractions and the protease enzymes themselves, as these compounds will be collected and discarded in the retentate. Further purification techniques can also be applied including chromatographic techniques, mainly reversed-phase high-performance liquid chromatography (RP-HPLC) and ultra-performance liquid chromatography (UPLC) depending on the level of purity desired in the final product. Previous studies generating bioactive peptides from the macroalga *Ulva* spp. applied MWCO followed by preparative RP-HPLC at wavelengths of 214 nm, to detect peptide bonds, and 280 nm, indicative of the presence of aromatic amino acids [64]. These and other purification strategies to isolate bioactive peptides from algae have been recently reviewed in detail by Lafarga et al. [62].

## 3. Biological Activities and Modes of Action of Algal Peptides

The generation of bioactive peptides is gaining momentum due to the wide range of biological properties attributed to these compounds that have been extensively reviewed [34,62]. Thus, this section will briefly mention a few examples of the anti-hypertensive, antioxidant, and anti-cancer activities from algae described in the recent scientific literature, also focusing on relating these described activities to their proposed mechanism of action and tools used for these analyses.

### 3.1. Antihypertensive Peptides

Cardiovascular disease (CVD) is one of the leading causes of mortality in the world today and hypertension is a significant risk factor for CVD. The regulation of blood pressure is mainly maintained by the renin angiotensin pathway. Briefly, the renin angiotensin system works by the secretion of renin into the blood system from the kidneys. Renin then binds the peptide angiotensinogen and forms angiotensin I. The angiotensin converting enzyme (ACE) binds and cleaves angiotensin I and transforms it into the highly potent vasoconstrictor angiotensin II, thus increasing blood pressure [65].

Fitzgerald et al. [66] extracted protein from the macroalgae *P. palmata* and performed an enzymatic hydrolysis with papain, identifying within the hydrolysate the peptide IRLIIVLMPILHA which potently inhibited the enzyme renin. Moreover, when this peptide sequence undergoes an in vitro digestion process, the gastrointestinal enzymes cleaved the peptide resulting in the production of the di-peptide IR with high anti-renin activity. In a follow up in vivo study using spontaneously hypertensive rats (SHR) and dosing with oral gavage, captopril reduced the blood pressure by 29 mm Hg, while the *P. palmata* hydrolysate reduced it by 34 mm Hg and IRLIIVLMPILHA peptide showed a reduction of 34 mm Hg [67].

ACE is a highly druggable target and several widely prescribed antihypertensive agents (e.g., captopril) are ACE inhibitors [68]. These inhibitors function by preventing the ACE-mediated conversion of angiotensin I into angiotensin II, preventing an increase in blood pressure. Captopril is a proline-based synthetic analog of a peptide present in snake venom that is a competitive inhibitor of ACE [69]. However, drugs like captopril, enalapril and lisinopril have serious adverse side effects that include dry cough, skin rashes, renal failure, and congenital malformations amongst others [70,71]. Thus, there is a growing interest in isolating new peptides with ACE inhibitory activity from natural sources, including those in food [72]. 

Multiple peptides with ACE inhibitory properties have been isolated from protein extracts from the microalgae *C. vulgaris* and *A. platensis* followed by enzymatic hydrolysis with pepsin [73]. In vivo tests evaluating the efficiency of peptides in SHR revealed that the oral administration of the tetrapeptide IAPG—isolated from *A. platensis*—resulted in a decrease in systolic blood pressure by approximately 50 mm Hg within 1 h of its ingestion [73]. The tripeptide FAL—isolated from *Chlorella*—was less potent in the SHR model, leading to a decrease of approximately 40 mm Hg within 2 h of ingestion. Moreover, the physiological effects of both IAPG and FAL in the SHR were sustained for 4 h post-ingestion [73].

Using a similar approach, Sun et al. [74] prepared hydrolysates from the macroalga *Ulva intestinalis* protein using trypsin, pepsin, papain, α-chymotrypsin and alcalase, and determined the in vitro activity of these hydrolysates when inhibiting ACE. The authors determined that trypsin-derived hydrolysates had the greatest inhibitory effect and identified the peptides FGMPLDR and MELVLR as those responsible for this effect. The authors also performed molecular docking with AutoDock 4.2 to reveal that while both peptides were bound to the active site, the mode of binding was different [74]. FGMPLDR interacted with Glu123, Ala354, Ala356, Glu384, and Arg522 and in particular with Ala354 and Glu384 which are both present in the S1 pocket of ACE, interacting with a well-known ACE inhibitor, lisinopril. In contrast, MELVLR was predicted to interact with Asn70, Glu143, Gln281, His383, and Lys511, with Gln281 and Lys511 of particular importance and located in the S2 pocket of the active site of ACE [74].

**Figure 1:**
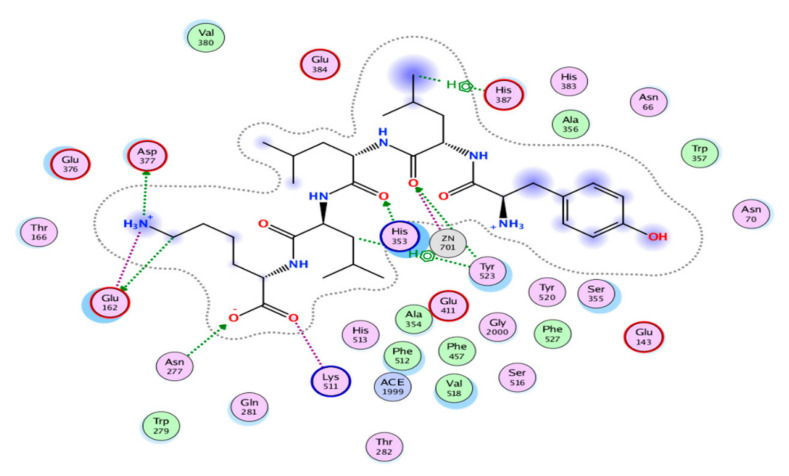
Automated molecular docking of the peptide YLLLK at the ACE active site. ACE hydrophobic residues are represented in green, positively charged residues in blue, and negatively charged residues in red; hydrogen bonds are purple arrows, polar residues are in turquoise color, and other residues and the zinc atom are represented automatically. Image obtained from Zarei, Abidin, Auwal, Chay, Abdul Haiyee, Md Sikin and Saari [75] originally published by MDPI.

To our knowledge, there are not many studies with bioactive peptides from algae linking their structure to a proposed mechanism of action. Zarei et al. [75] studied the ACE inhibitory mechanism of action of the bioactive peptides YLLLK, WAFS and GVQEGAGHYALL identified from palm kernel cake. The authors noted concentration-dependent effects on enzyme inhibition, consistent with the presence of more than one binding site for the peptides and potentially multiple modes of inhibition [75]. Moreover, differences were appreciated in the way that these peptides achieved their activity, as some peptides showed variable degrees of degradation upon pre-incubation with ACE. The authors concluded that the peptide YLLLK acted as a competitive inhibitor and exhibited a higher number of total interactions with ACE compared to the other two peptides [75]. The action of the peptide YLLLK at the ACE active site visualized using molecular docking is represented in Figure 1. Ni, Li, Liu and Hu [69] determined that the ACE inhibition of the yeast peptide TPTQQS was caused by non-competitive interactions by displacing the Zn cofactor from the active site of the enzyme so the reaction cannot occur. The majority of the peptide is attached outside of the active site; however, the tail end of the peptide containing the serine 6 residue is what comes into contact and sequesters the zinc ion by forming a coordination bond with it [69].

### 3.2. Antioxidant Properties

Free radicals are short-lived and highly reactive chemical species that contain unpaired electrons [76]. Although reactive oxygen species (ROS) are formed during normal metabolic processes, they may also be formed due to exposure to exogenous factors such as ionizing radiation and UV light [77]. Free radicals can oxidatively modify nucleic acids, proteins, lipids, and sugars. An increased presence of these modified forms has been associated with an increased risk of various human diseases (e.g., cancer, Alzheimer’s disease) [78]. Antioxidant mechanisms are present in most organisms and act to reduce or eliminate the levels of common ROS [77]. While many of the most effective mechanisms are enzyme-based (e.g., superoxide dismutase and catalase). Many small molecules (e.g., vitamin C, glutathione) also play a role in maintaining the overall redox balance in the cell [79]. While both natural and synthetic antioxidants have been added to foodstuffs, the potential toxicity of synthetic antioxidants, e.g., GHT has prompted the exploration of the use of peptides as antioxidant agents [77,79,80]. It is generally accepted that large peptides have less radical scavenging or antioxidant potential than small peptides [81]. Peptides have been reported to exert their antioxidant activity through direct metal chelation, ROS scavenging and inhibition of lipid peroxidation cascades [82].

A peptide from the microalgae *Isochrysis zhanjiangensis* has shown to have potent antioxidant capabilities towards alcohol-induced injury in cultured liver hepatoma cells (HepG2) [83]. This peptide was produced by in vitro gastro digestion using pepsin, trypsin and chymotrypsin resulting in the active sequence of NDAEYGICGF [83]. The treatment of cells with this peptide resulted in increased levels of the enzymes superoxide dismutase and glutathione. The antioxidant capabilities of the peptide appear to be related to a combination of the following factors: its molecular weight, hydrophobic amino acids (A, G, I) and aromatic amino acids (F, Y) in the sequence [83]. Other antioxidant peptides identified from *C. vulgaris* include VECYGPRPQF, which showed antioxidant capacity 26-fold higher (197 ng/mL Trolox equivalent) than trolox when tested by ORAC [84]. This peptide was found to slow oxidation by up to 10-fold compared to the control (PBS). The authors hypothesized that the Cu^2+^ chelating properties of this peptide were likely the main mechanism of action of antioxidant activity [84]. 

### 3.3. Anti-Cancer Properties

Peptides, due to their small size and chemical nature, can penetrate cell membranes without a build-up of toxic levels as seen with protein/antibodies. These compounds have shown high affinity and specificity while having low interactions with other medical treatments. However, there are limitations to overcome for their use, mainly related to the process of delivery of the peptide, as these compounds have regularly low bioavailability when taken orally, resulting in rapid clearance of the peptides. Peptides also have low levels of activity when compared to traditional drug treatments for cancer [85]. However, the peptide treatments have multiple problems mainly associated with the lack of specificity of the drugs that are not able to differentiate between carcinogenic and healthy cells. Moreover, when the chemotherapeutic agents are bound to a transport molecule, the breakage of these bounds can also reduce the efficacy of the peptides. Furthermore, the ability we have to currently treat multiple cancers is also dependent on the resistance of the cancer to the chemotherapeutic agents, which is a growing problem [86].

Limited studies are available on the anti-cancer properties of peptides derived from algae. Sheih, Fang, Wu and Lin [84] hydrolyzed protein by-products from the industrial processing of *C. vulgaris* and identified the peptide VECYGPNRPQF as an anti-proliferative. This peptide only had anti-proliferative effects on the human gastric cancer cell line AGS, but not on the other cell lines studied including human normal lung cell WI38, human colon adenocarcinoma cells C2BBel, human hepatoblastoma cell lines HepG2, human cervical epithelioid carcinoma cells Hela, and mouse BALB/c macrophage RAW 264.7 cells. The authors hypothesized that this peptide could have specific anti-cancer activity when treating certain tumor cells [84]. The peptide halted the cell cycle where the cell is given the chance to be either repaired by the TP53 mechanism or undergo apoptosis [87]. Moreover, the number of cells in the G1 phase decreased, while the Sub G1 phase category increased, indicating that the cells entered an apoptotic pathway following 48 h of incubation [84].

Anti-proliferation effects have been recorded from peptides produced by trypsin hydrolysis of proteins from *Porphyra haitanesis* [88]. The peptides generated were tested using five human cancer cell lines tested: MCF-7 (breast cancer), HepG2 (liver cancer cells), SGC-7901 (gastric cancer), A549 (lung cancer) and HT-29 (colon cancer), using the chemotherapeutic drug fluorouracil (5-FU) as a control. Four fractions (by size kDa) were obtained from the hydrolyzed peptides, and the peptide VPGTPKNLDSPR was reported as that with the highest antiproliferative activity, even significantly more potent than 5-FU in a HepG2 cell model [88]. Mechanically, this peptide appeared to interfere with the cell cycle and promoted apoptotic cell death in HepG2 and MCF7 cells [88].

The efficacy of several algal peptides in oncological treatments and their mechanisms of action have been elucidated. Kahalalides are an assortment of depsipeptides ranging in size from 31 carbon tripeptides to 75 carbon tridecapeptides. The peptide was firstly found in the mollusk *Elysia rufescens* and it was further discovered to be present in the algae *Bryopsis pennata* consumed by the mollusk and acting as a defense mechanism against predators [89]. Amongst all the Kahalalides, the one showing the most promise in cancer treatment is the largest peptide, Kahalalide F (KF) C_75_H_124_N_14_O_16_ [90,91]. KF has shown its potential benefits for cancer treatment in both in vitro and in vivo preclinical trials. Suárez, González, Cuadrado, Berciano, Lafarga and Muñoz [90] studied the mechanism of action of KF to determine its cytotoxic action against neoplastic cells. The authors used prostate (PC3, DU145, LNCaP) and breast cancer (SKBR-3, MCF7, BT474, MDA-MB-231) cell lines. The IC_50_ of KF for all the cell lines was around 0.3 µM, except in the case of PC3 which was 0.07 µM. Moreover, the authors also showed that the cytotoxic response of KF appears quickly, within 15 min. KF’s mechanism of action differs from that of other antineoplastic drugs as it does not cause apoptosis, but generated an ATP depletion and swelling of the cells or oncosis [90,92]. KF has a similar mechanism of action to that of maitotoxin, a peptide that causes oncosis as its action linked to the function of the calcium ion channels of the cells [92]. Although maitotoxin is one of the most potent marine peptides known to date, its action is non-selective, and it is responsible for a particular human intoxication syndrome, namely ciguatera fish poisoning [93]. In this regard, KF could be a better fit for anti-cancer treatments as it has displayed tumor-selective properties in testing and has low toxicity [94].

## 4. Application of Novel Bioinformatic Tools 

Bioinformatic tools are routinely used in peptide and protein analysis. In the context of the production of peptides via protein hydrolysis, online peptide cutters can simulate the cleavage of peptide sequences by various enzymes [95]. Toxicological and allergenic properties may also be predicted, using tools such as ToxinPred [96] and AllerTOP [97], respectively. Moreover, the BIOPEP database [98] compiles the reported activities of various peptide sequences. In silico methods being used to identify bioactivity in peptides include the use of quantitative structure–activity relationships (QSAR) and molecular docking, aiming to identify the mechanism of action underlying the biological functions of these peptides. 

### 4.1. QSAR

One of the main challenges arising when producing a hydrolysate from a mix of proteins is to be able to elucidate which one of the multiple structures present in the hydrolysate is responsible for the biological effects appreciated during in vitro or in vivo tests and establish their mechanisms of action. When studying antioxidant peptides and only considering dipeptides, there can be potentially 400 different structural combinations accounting for all the possible combinations of 20 amino acids. However, when studying oligopeptides (2–20 amino acids in length) [99], this variability can reach levels of over 1.07 × 10^39^ possible structural combinations and thus, the use of bioinformatic methods, such as QSAR, can support the identification of bioactive peptides [100].

**Figure 2:**
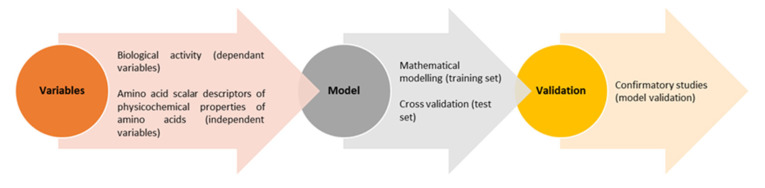
Process flow of QSAR applied to bioactive peptides. Content of the image adapted from Nongonierma and FitzGerald [101].

QSAR is an in silico method which takes peptides and their biological activity from pre-existing databases, such as BIOPEP, aiming to understand the link between these structures and their activity towards different biological targets [101]. The process flow of QSAR is represented in Figure 2.

When sourcing bioactive peptides of interest in a QSAR model it is important to note that some peptides work in different ways of inhibiting their targets, such as competitive, non-competitive, and un-competitive ways. If a particular peptide library lacks the specific type of inhibitory action of these peptides then it completely skews the ability of the QSAR to estimate IC_50_ [101]. These peptides are used to create a model which aims to identify the key commonalities of the structure and composition of these peptides and link their composition to a bioactivity of interest. A portion of the peptides from the data set is randomly selected and left out of the training set; these are the test set and will be used at a later stage of the process. These peptides are used to identify the causative structure that allows for these interactions to occur, allows the identification of peptides with the most advantageous structural features, and establishes prediction scores for these structures [101]. These known peptides teach the software what to look for when unknown peptides are plugged into the equation to be identified and the peptides used to create the QSAR model should be a similar size to the peptides being analyzed [101].

Kumar et al. [102] researched novel ACE inhibitory peptides and the massive variation in these results due to the variable length of peptides with ACE inhibitory activity. This author chose the libraries where peptides with the same mechanisms of action for a particular bioactivity were classed by size, and QSAR models should be produced for each class to increase the accuracy of the results. The authors classified ACE inhibitory peptides as <3 amino acids, small peptides as 4–6 amino acids, medium peptides as 7–12 amino acids and large peptides as >12 amino acids [102]. 

Different scales and descriptors can be used to accurately define the features that make a certain peptide bioactive. The correct choice of descriptors is important as an excessive number of descriptors will cause background noise, causing an overfitting of data and loss of predictive accuracy [103]. These descriptors are usually physiochemical characteristics, such as the scale described by Hellberg et al. [104] which uses 29 physiochemical descriptors to analyze the amino acids. The authors grouped these descriptors into three main components known as the 3 Z approach which explains hydrophilicity (Z_1_), steric properties (Z_2_) and electronic properties (Z_3_) [104]. This approach was improved further by Sandberg et al. [105] when characterizing 87 amino acids by adding two further components—Z_4_ and Z_5_—to describe other properties of the amino acids, such as heat of formation, electronegativity and electrophilicity. 

From these rankings, multiple regression models can be performed to evaluate the bioactive potency of peptides on the basis of the interaction between the peptide and its target. These tests are normally performed against a positive control, such as glutathione for antioxidant peptides, and if the tested compounds score similar or even higher than the control, those compounds may have a better potential for in vitro testing [101]. After the model has been run and the IC_50_ predicted, these data require validation using the test set of peptides that were set aside at the beginning. Moreover, the models will have to be confirmed by testing the highest-ranking peptides against a laboratory-based assay and ensuring that the IC_50_ predicted by the model matches the experimental data [101]. If the model predicted the activity accurately, then the peptides which are of interest to be tested will be ranked by potency, synthesized, and experimentally tested to compare the results with those of the QSAR model [101]. 

The third step in the process of making a QSAR is the selection of a mathematical model to relate the physiochemical characteristics and position of the amino acids in the C and N terminus of the tested peptides with those of the peptides with known bioactivity. The models chosen are usually partial least square regression (PLSR), iterative double least square (IDLS), artificial neural networks (ANN) and multiple linear regression (MLR). When using these models, it is important that the model chosen accounts for whether the peptides being screened for activity will be of the same length as those described in the training set or if they account for peptides with a variety of different sizes [100,101].

### 4.2. Molecular Docking

Molecular docking is a stage to further identify the interactions between the target and substrate, complementing the QSAR modeling as it will provide three-dimensional interactions between the ligand; in this case, the peptide and the target to which the peptide is binding to help to understand further their inhibitory effects [106]. 

**Figure 3:**
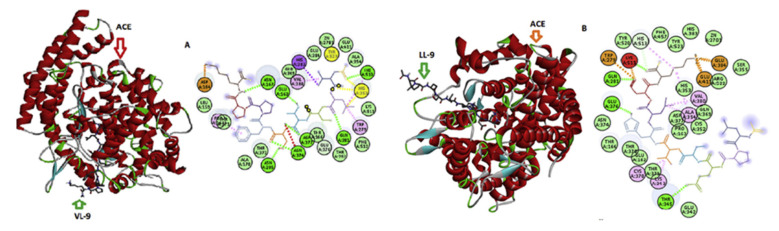
Representation of the molecular docking results (3D and 2D) of the ACE-inhibitory peptides VL-9 (**A**) and LL-9 (**B**). Color codes are as follows: blue (Van der Waals bonds), orange (salt bridge) and green (conventional hydrogen bond). Image originally published by Mirzaei, Mirdamadi, Ehsani and Aminlari [107] in Elsevier.

Angiotensin-converting enzyme (ACE) has been a focus of molecular docking studies in relation to cardiovascular diseases to further understand the action of peptides working within its domains. Mirzaei et al. [107] used the crystal structure of human ACE complexed with inhibitor lisinopril as a template for docking studies using the software HADDOCK (see Figure 3). The authors removed all water molecules and the inhibitor from the structure while retaining the zinc and chloride atoms in their active site before proceeding with the docking [107]. As previously stated, a disadvantage of QSAR is that it can be dependent on the amount of information granted to it by the database. An example of this is not identifying if the peptide is competitively binding to the active site or if it is having another effect on the enzyme in its entirety. Using molecular docking on the highest-ranking peptides from QSAR will show their overall binding affinity to the active site; this will hopefully mitigate any problems caused by the lack of information from these databases before synthesizing the highest-ranked peptides and final laboratory confirmatory testing.

## 5. Opportunities and Challenges

There are huge market opportunities for algae as a source of protein due to the environmental benefits [108] associated with its production as well as their untapped potential as source of food and food ingredients for the growing world’s population. However, there are still challenges, mainly related to the creation of optimum, reproducible, and sustainable protein extraction processes, mainly limited by the variable composition of the biomass as well as the presence of rigid cell walls of a variable chemical nature. Moreover, all the pre-treatments of the biomass, and the new emerging technological treatments, will have to demonstrate its economic viability in order to be adopted by industry, allowing to scale-up production and expand the use of these approaches.

In addition, further studies evaluating the activity and the chemical structure of peptides will be necessary to build upon current peptide libraries. The choice of peptide library is extremely important for the validity of the QSAR for testing unknown peptides and molecular docking studies. There are massive opportunities in the search for new peptide alternatives to be used as nutraceuticals with fewer adverse side effects than conventional treatments for multiple diseases. However, further studies and clear mechanisms of action have to be elucidated for these applications to achieve their potential.

## Figures and Tables

**Table 1:** Characteristics of proteases used for the generation of bioactive peptides.

Enzyme Name	Type of Enzyme	pH Range	Temperature	Cleavage Preference	References
Trypsin	Serine protease	7.8	37–42 °C	Positively charged amino acids; R and K	[35]
Chymotrypsin	Serine protease	7.8	37–42 °C	Hydrophobic amino acids; Y, F and W	[36]
Pepsin	Aspartic protease	1.25–2.5	37–42 °C	Positively charged amino acids; R and K	[37]
Alcalase	Serine endopeptidase	6.5–10	60–75 °C	Broad range specificity however, propensity for cleaving aromatic amino acids	[38,39,40]
Papain	Cysteine endopeptidase	6–7	65 °C	Broad range specificity, cleaving peptide bonds of basic amino acids, L or G. Papain will not accept V at position 1 and at position 2 prefers large hydrophobic amino acids	[41]
Bromaline	Cysteine endopeptidase	4.5–8	35–55 °C	Broad range specificity with preferred cleavage site at the C terminus of K, A, Y and G	[42]
Protamex	Mixture of endo- and exo-proteases from *Bacillus* sp.	6–9	30–65 °C	Broad cleavage range as it is a mixture of proteases	[43,44]
Elastase	Serine protease	9	37 °C	Preferred cleavage at the C-terminus of A, V, S, G, L and I	[45]
Thermolysin	Metalloproteinase	5–8.5	65–85 °C	Preferred cleavage at the N-terminus of F, V, I, L, M and A	[46]

## Data Availability

Not applicable.
